# Theory of mind in users of anabolic androgenic steroids

**DOI:** 10.1007/s00213-020-05603-y

**Published:** 2020-07-05

**Authors:** Anja Vaskinn, Lisa E. Hauger, Astrid Bjørnebekk

**Affiliations:** 1 Norwegian Centre for Mental Disorders Research, Division Mental Health and Addiction, Oslo University Hospital, PO Box 4956, Nydalen, 0424 Oslo, Norway; 2 Institute of Clinical Medicine, University of Oslo, PO Box 1171, Blindern, 0318 Oslo, Norway; 3 The Anabolic Androgenic Steroid Research Group, National Advisory Unit on Substance Use Disorder Treatment, Division Mental Health and Addiction, Oslo University Hospital, PO Box 4959, Nydalen, 0424 Oslo, Norway

**Keywords:** Mentalizing, Mindreading, Testosterone, Dependence, Social cognition

## Abstract

**Rationale:**

Anabolic androgenic steroids are used to improve physical performance or increase lean muscle mass. About one-third of users develop a dependency syndrome, which is characterized by elevated rates of psychopathology, cognitive impairments, and aggressive and antisocial behaviors. The mechanisms behind these intra- and interpersonal problems are not known.

**Objective:**

To examine theory of mind (ToM), i.e., the ability to infer the mental state of others, in users of anabolic androgenic steroids. Reduced ToM may be one factor underlying the interpersonal problems that have been reported with prolonged use of anabolic androgenic steroids.

**Methods:**

The Movie for the Assessment of Social Cognition (MASC) was used to assess ToM. Study participants were male/female weightlifters who used anabolic androgenic steroids (AAS, *n* = 34/9), who were dependent on anabolic androgenic steroids (AASdep, *n* = 44/7), and a non-using weightlifting comparison group (WLC, *n* = 69/16).

**Results:**

Analyses of variance showed that the AASdep group performed significantly worse than the WLC group, for all MASC measures (total ToM, cognitive ToM, affective ToM, overmentalizing/undermentalizing errors). Sex and sex x group interaction effects were non-significant.

**Conclusions:**

Male and female weightlifters who were dependent on anabolic androgenic steroids had impaired ToM. Their reduced social cognition may be one contributing factor to the elevated rates of antisocial behavior reported in this population.

## Introduction

Human social behavior is a result of numerous interacting factors. Among these are the “social” hormones oxytocin and testosterone. Whereas oxytocin, aka the “love” hormone in popularized jargon, is involved in affiliative behavior, bonding, and care (Macdonald and Macdonald 2010), testosterone has often been linked to aggression (Montoya et al. 2012). The complete picture, however, is less straightforward. Although both hormones influence human social behavior, the effect depends on the situation (van Honk et al. 2011a). Oxytocin can have antisocial effects, if antisociality provides benefits for offspring, in-group members, or reproductive partners (Beery 2015). Testosterone has been proposed to drive motivation for seeking and maintaining social status, and not for aggression per se (Eisenegger et al. 2011). Interestingly, single dose administration of testosterone has been shown to lead to suppression of facial mimicry (Hermans et al. 2006), reduced ability to infer the emotions and intentions of others (Van Honk et al. 2011b), and to reduced trust (Bos et al. 2010). When competing for social status, there may be clear disadvantages to trusting, caring, or empathizing with the rival (Eisenegger et al. 2011). The reduced social cognition seen in these studies may therefore be beneficial for the individual in the situation where (s)he seeks social status. However, reduced social cognition also has clear disadvantages. Understanding and empathizing with the feelings of another is a key ingredient of mutually satisfying relationships. Indeed, social cognitive impairment is an important predictor of reduced social functioning in various mental disorders (Fett et al. 2011; Vlad et al. 2018; Halversen et al. 2019), including substance use disorders (Preller et al. 2014).

Administration of testosterone-like substances far beyond a single dose is at the core of one substance use disorder, namely anabolic steroid dependence. Anabolic androgenic steroids (AAS) comprise testosterone and its synthetic derivatives. Due to their anabolic effect, they are used by athletes, bodybuilders, and recreational athletes, to improve physical performance or increase lean muscle mass. Moreover, due to profound masculinizing features of AAS, they are foremost used by men (Sagoe et al. 2014), although use is also seen among female bodybuilders or fitness athletes (Gruber and Pope Jr 2000). When used to increase muscle mass (Parkinson and Evans 2006), they are often administered in doses that exceed the natural male production by 5–100 times (Brower 2002). These supraphysiological doses cause large alterations to the hormonal system, which in turn likely increase the hormonal effects on cognition, mood, and behavior.

It is estimated that about a third of AAS users develop a dependency syndrome, characterized by a maladaptive pattern of AAS use, which is maintained despite substantial negative consequences (Brower 2002; Kanayama et al. 2009a). Among adverse side effects reported for prolonged AAS use are reduced social (Hauger et al. 2019) and non-social cognition (Kanayama et al. 2013; Heffernan et al. 2015; Bjørnebekk et al. 2019; Hauger et al. 2020), increased psychiatric symptoms (Kanayama et al. 2008; Oberlander and Henderson 2012), and medical problems (Oskui et al. 2013), including damage to the cardiovascular system (Kanayama et al. 2008; Far et al. 2012; Baggish et al. 2017; Rasmussen et al. 2018) and infertility (de Souza and Hallak 2011). AAS users who develop a dependency syndrome report elevated rates of psychopathology (Kanayama et al. 2009b), psychological distress, and executive dysfunction (Hauger et al. 2020) compared with non-dependent AAS users. Furthermore, AAS dependence is associated with higher levels of involvement in aggressive and antisocial behaviors (Copeland et al. 1998, 2000). The exact mechanisms behind these elevated rates in dependent AAS users are unknown. Administration of testosterone has been shown to increase aggression (Montoya et al. 2012), which is one of the side effects that are commonly linked to AAS use (Beaver et al. 2008; Pope Jr and Katz 1994; Yates et al. 1992). However, these are complex relationships, probably including a number of mediating factors. It is quite possible that the negative outcomes seen in prolonged use/dependency of AAS are attributable to premorbid factors responsible for both the dependency and increased interpersonal problems.

AAS have, as testosterone, been associated with social-emotional processes. We have previously shown that AAS dependence is associated with impaired social cognition, more specifically the ability to recognize emotions in moving bodies (Hauger et al. 2019). One definition of social cognition refers to it as the “mental operations that underlie social interactions, including perceiving, interpreting and generating responses to the intentions, dispositions and behavior of others” (Green et al. 2008). This is a wide definition, and the ability to recognize emotions displayed by others, or emotion perception, is one of several social cognitive domains (Pinkham 2014). In our previous study, emotion perception was assessed using basic point-light stimuli (Hauger et al. 2019). This social cognitive process entails decoding human movement in simple stimuli and can be considered a bottom-up process (Ochsner 2008). Another social cognitive domain, mentalizing/theory of mind (ToM), on the other hand, is a top-down deductive process (Ochsner 2008), involving the capacity to infer and interpret the mental state of others (Brüne and Brüne-Cohrs 2006). To our knowledge, ToM has thus far not been investigated in AAS users. Given studies that have found ToM impairments in other substance using populations (Bora and Zorlu 2017; Sanvicente-Vieira et al. 2017), one might suspect that it may be reduced also in AAS dependence. Support for such a hypothesis also comes from research in developmental psychology suggesting that androgens may influence on ToM. One study found that fetal testosterone levels had an impact on empathy in 6–8 year olds (Chapman et al. 2006). This finding was corroborated in more recent work, where it was shown that the level of prenatal androgens could exert an influence on ToM (Khorashad et al. 2018). One speculation is that impaired ToM could be one mechanism behind the elevated rates of interpersonal problems in AAS dependence (Copeland et al. 1998, 2000). In this study, we examine ToM in users of AAS, with or without dependence, compared with weightlifting control participants. We hypothesize reduced ToM performance in AAS users with dependence.

## Methods

### Participants

Study participants consisted of male (*n* = 147) and female (*n* = 32) weightlifters > 18 years of age with either (a) current or previous use of AAS or (b) no previous or current use of AAS or other doping substances. Participants were recruited via social media (Facebook) and online forums and webpages targeting people interested in heavy weight-training or bodybuilding. Additionally, posters and flyers were distributed in selected gyms in Oslo, Norway, and some recruitment took place through snowball sampling. Prior to participation, all participants received a brochure with a description of the study, and written informed consent was collected. The study was conducted in accordance with the Declaration of Helsinki and received ethical approval from the Regional Committee for Medical and Health Research Ethics in South-Eastern Norway (2013/601). Participants received NOK 1.000 (≈ $125) as compensation for taking part in the study.

Exclusion criteria were a history of severe head injury with loss of consciousness for > 1 min, a neurological disorder (e.g., history of diagnosed stroke, brain tumor, Parkinson’s disease, or epilepsy), or IQ < 80. We were interested in AAS use across biological sex. As AAS are used almost exclusively by males (98%), different inclusion criteria were applied for the two sexes in order to secure the inclusion of females. Male AAS users were included if they had > 1 year of cumulative AAS exposure, when summarizing periods on cycle. Female AAS users were included if they had used AAS for at least one cycle. In total, 192 participants (159 males/33 females) were eligible for participation and enrollment in the overall study (Bjørnebekk et al. 2019), but 5 did not meet the inclusion criteria. In addition, 8 participants were not included in the current study due to missing data for the main study variable. The male sample is largely overlapping with the one described in our previous work which included only males (Hauger et al. 2019, 2020; Bjørnebekk et al. 2019). Three groups of weightlifters participated. The weightlifting control participant group (WLC) consisted of 85 individuals (69 males/16 females). There were 43 individuals in the non-dependent AAS group (AASnondep) (34 males/9 females). The dependent AAS group (AASdep) had 51 participants (44 males/7 females). The subdivision into AASdep and AASnondep was based upon AAS dependence criteria (Kanayama et al. 2009c). These are described in the next section.

Sex differences appeared, unsurprisingly, for weight and height, in the expected direction (males > females). Furthermore, the three groups differed significantly for length of education and IQ. The AASdep group had the shortest education and the lowest IQ. Significant group differences were also present for drug use, with AAS dependent males showing particularly elevated scored (one standard deviation above the normative mean). For details concerning demographics and alcohol/drug use, see Table 1.

**Table 1:** Demographics of individuals using anabolic androgenic steroids (AAS), individuals with anabolic androgenic steroid dependence (AASdep), and of weightlifting control participants (WLC)

	WLC (*n* = 85)		AAS (*n* = 43)		AASdep (*n* = 51)		Statistics
	Females *n* = 16 Mean (SD)	Males *n* = 69 Mean (SD)	Females *n* = 9 Mean (SD)	Males *n* = 34 Mean (SD)	Females *n* = 7 Mean (SD)	Males *n* = 44 Mean (SD)	
Age	28.4 (4.5)	31.8 (9.5)	28.7 (7.5)	33.2 (8.3)	34.0 (7.4)	33.4 (8.6)	Group: *F* = 1.5, *p* = 0.229 Sex: *F* = 1.9, *p* = 0.169 G x S: *F* = 0.6, *p* = 0.528
Education	16.0 (2.1)	15.8 (2.7)	14.6 (1.5)	14.5 (2.7)	14.2 (2.7)	13.9 (2.2)	*Group: F = 5.5, p = 0.005* Sex: *F* = 0.1, *p* = 0.707 G x S: *F* = 0.0, *p* = 0.979
WASI IQ	108.0 (8.1)	113.0 (9.4)	101.0 (16.2)	107.8 (11.5)	102.7 (15.2)	102.6 (11.5)	*Group: F = 5.4, p = 0.005* Sex: *F* = 3.0, *p* = 0.085 G x S: *F* = 0.7, *p* = 0.495
Height (cm)	167.2 (7.8)	180.7 (6.9)	165.4 (6.5)	179.7 (6.1)	167.7 (6.8)	181.3 (7.7)	Group: *F* = 0.6, *p* = 0.556 *Sex: F = 90.4, p < 0.001* G x S: *F* < 0.1, *p* = 0.970
Weight (kg)	65.5 (9.8)	90.5 (14.0)	64.3 (9.8)	94.1 (12.9)	68.0 (10.2)	99.2 (13.9)	Group: *F* = 1.5, *p* = 0.222 *Sex: F = 112.1, p < 0.001* G x S: *F* = 0.6, *p* = 0.563
Activity, *n* (%)							
Body building	7 (44%)	6 (9%)	2 (22%)	9 (26%)	3 (43%)	14 (32%)	*Group x activity:* *x*^*2*^ *= 23.7, p = 0.003*
Weightlifting	2 (13%)	18 (26%)	0 (0%)	1 (3%)	1 (14%)	0 (0%)	
Combat	1 (6%)	4 (6%)	2 (22%)	4 (12%)	0 (0%)	5 (11%)	Sex x activity: *x*^2^ = 6.2, *p* = 0.182
Recreational	5 (31%)	31 (45%)	5 (56%)	15 (44%)	3 (43%)	20 (46%)	
Other	1 (6%)	9 (13%)^1^	0 (0%)	5 (15%)	0 (0%)	5 (11%)	
Alcohol use T	55.9 (5.3)	60.0 (7.0)^2^	58.1 (4.4)	56.6 (7.0)^3^	58.0 (7.5)	57.7 (7.1)^4^	Group: *F* = 0.1, *p* = 0.922 Sex: *F* = 0.3, *p* = 0.602 G x S: *F* = 1.8, *p* = 0.164
Drug use T	50.3 (1.3)	51.6 (6.8)^2^	52.9 (5.4)	54.4 (8.6)^3^	54.9 (8.3)	60.1 (15.2)^5^	*Group: F = 3.9, p = 0.023* Sex: *F* = 1.8, *p* = 0.181 G x S: *F* = 0.4, *p* = 0.690

Entries in italics means that a result is statistically significant

^1^One male in the weightlifting group had missing value for this variable

^2^*n* = 62 due to missing data

^3^*n* = 28 due to missing data

^4^*n* = 36 due to missing data

^5^*n* = 37 due to missing data

### Clinical measures

The presence of lifetime AAS dependence was evaluated in a standardized clinical interview using a version of the Structured Clinical Interview for DSM-IV (SCID) (First et al. 1996). This version is based upon the standard substance-dependence criteria of DSM-IV, but has been modified and adapted to apply specifically to AAS dependence (Kanayama et al. 2009c), preserving adequate psychometric properties (Pope et al. 2010). AAS dependence was considered to be present if participants had a maladaptive pattern of AAS use causing clinically significant impairment or distress, manifested by three (or more) of the DSM-IV criteria (Kanayama et al. 2009c) reported in the same 12-month period. The AASdep group included users with a lifetime history of AAS dependence, both current and previous. The two substance use scales of the Achenbach System of Empirically Based Assessment (ASEBA) and Adult Self-Report (ASR) questionnaire (Achenbach and Rescorla 2003) were used to provide measures of use of illegal drugs during the past 6 months. There was some missing data for males (WLC: 5, AAS: 6, AASdep: 8–9). Significant group differences appeared for the drug use subscale, with the AASdep showing the highest rates, see Table 1 for details. Characteristics related to use of AAS in the two AAS groups are shown in Table 2.

**Table 2:** Characteristics related to use of AAS of the non-dependent (AAS) and dependent (AASdep) subgroups

	AAS (*n* = 43)	AASdep (*n* = 51)	Statistics	
	Mean (SD)	Mean (SD)	*t*	*p* value
Debut age of AAS use	23.3 (5.8)	21.6 (7.0)	1.25	0.214
Total years of AAS use	6.5 (5.4)	10.0 (6.1)	2.9	0.043
Estimated weekly AAS dose	1059.5 (1275.6)^1^	1287.2 (864.4) ^2^	0.99	0.322
	*n* (%)	*n* (%)	*X*^2^	*p* value
Current AAS use	27 (62.8)	32 (62.8%)	0.00	0.996
Physical side effects	33 (76.8%)	48 (94.1%)	5.91	0.015
Psychological side effects	26 (60.5%)	46 (90.2%)	11.50	0.001
Cognitive side effects	11 (25.6%)	31 (60.8%)	11.70	0.001

^1^*n* = 38 due to missing data

^2^*n* = 50 due to missing data

Relevant background information was captured by a semi-structured interview. The interview also covered details about AAS use, such as age of onset, administration patterns, years of use, weekly dosage, and experienced side effects. The AASdep group had used AAS for a longer time and reported significantly more physical, psychological, and cognitive side effects than the AAS group.

### Cognitive measures

IQ was assessed with the short version of the Wechsler Abbreviated Scale of Intelligence (WASI) (Wechsler 2007) which is comprised of the Vocabulary and Matrix Reasoning subtests. ToM was assessed with the Norwegian version (Fretland et al. 2015) of the Movie for the Assessment of Social Cognition (MASC) test (Dziobek et al. 2006). It is a 15-min movie depicting four characters in real-life interactions. The movie is paused 45 times and the test-taker instructed to answer questions concerning a character’s thoughts, emotions, or intentions. MASC is an ecologically valid test that provides not only an overall ToM score (MASCtot) but also information on cognitive (MASCcog) and affective (MASCaff) ToM. We categorized items as cognitive (thoughts, intentions) or affective (emotions) in accordance with previous research using the Norwegian version (Vaskinn et al. 2018). Furthermore, the test yields information on a person’s mentalizing style through its multiple choice response format. Every item has four response options. In addition to the correct answer, the response options correspond to overmentalizing (excessive attribution of mental state: MASCexc), undermentalizing (underinterpretation of mental state: MASCless), and no mentalizing (no attribution to mental state: MASCno).

### Statistical analyses

The overall research aim concerning group and sex differences in ToM performance was examined with a univariate analysis of variance (ANOVA) where MASCtot was entered as the dependent variable. Subsequently, two repeated measures ANOVAs (or mixed within-between-subjects ANOVAs) were conducted, for type of ToM (cognitive versus affective ToM) or ToM error types (overmentalizing, undermentalizing, or no mentalizing errors), respectively. In the first, a 2 × 3 × 2 repeated measures ANOVA, sex (males/females), and group (WLC, AAS, AASdep) were entered as between-subjects factors and the two MASC subscales (MASCcog, MASCaff) as within-subject factor. The second was a 2 × 3 × 3 repeated measures ANOVA where the only difference from the former was the entering of the three MASC error scores (MASCexc, MASCless, MASCno) as within-subject factor.

We conducted two types of follow-up analyses for MASCtot. First, background variables for which statistically significant differences appeared in initial group comparisons, i.e., IQ and drug use, (Table 1) were entered as covariates in separate follow-up analyses. Since education can be considered a proxy for IQ, it was not examined further. Second, possible differences between AAS users on and off cycle were examined through a univariate ANOVA with group (AAS versus AASdep) and cycle (on versus off) as independent variables. All analyses were conducted using The Statistical Package for the Social Sciences (IBM SPSS Statistics for Windows, Version 26.0, IBM Corp, Armonk, NY).

## Results

**Fig. 1:**
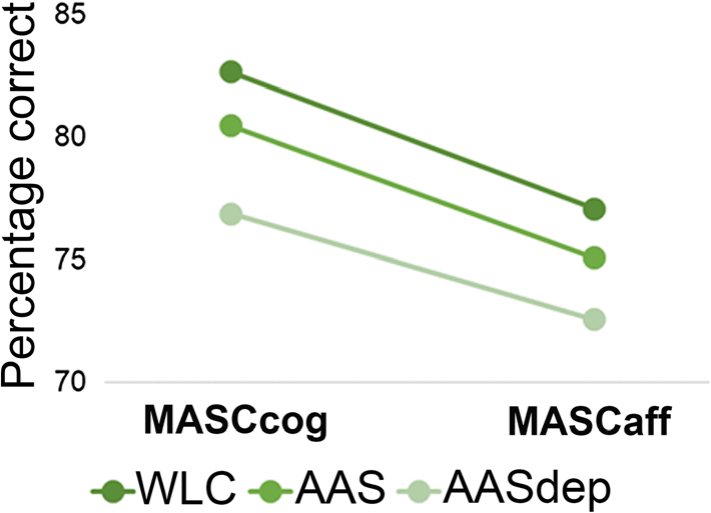
Cognitive (MASCcog) and affective (MASCaff) theory of mind performance in individuals using anabolic androgenic steroids (AAS), individuals with anabolic androgenic steroid dependence (AASdep), and in weightlifting control participants (WLC)

The overall univariate ANOVA yielded a significant main effect of group on MASCtot [*F*_(5,179)_ = 5.63, *p* = 0.004, *ŋ* = 0.06]. Post hoc Scheffe tests indicated that the WLC and AASdep groups differed significantly (*p* = 0.02). The main effect of sex [*F*_(5,179)_ = 0.04, *p* = 0.839, *ŋ* = 0.00] and the group x sex interaction effect [*F*_(5,179)_ = 0.85, *p* = 0.430, *ŋ* = 0.01] were non-significant. In the repeated measures ANOVA for ToM type, the main between-subject effect of group was significant [*F*_(2,173)_ = 5.19, *p* = 0.007, *ŋ* = 0.06]. Again, it was the WLC and AASdep groups that differed significantly (post hoc Scheffe, *p* = 0.002). Both the main effect of sex [*F*_(2,173)_ = 0.21, *p* = 0.645, *ŋ* = 0.00] and the group x sex interaction [*F*_(2,173)_ = 0.62, *p* = 0.538, *ŋ* = 0.00] effect were non-significant. The main within-subject effect of ToM type was significant [*F*_(1,173)_ = 730.48, Wilk’s Lambda = 0.19, *p* < 0.001, *ŋ* = 0.81] (see Fig. 1).

**Fig. 2:**
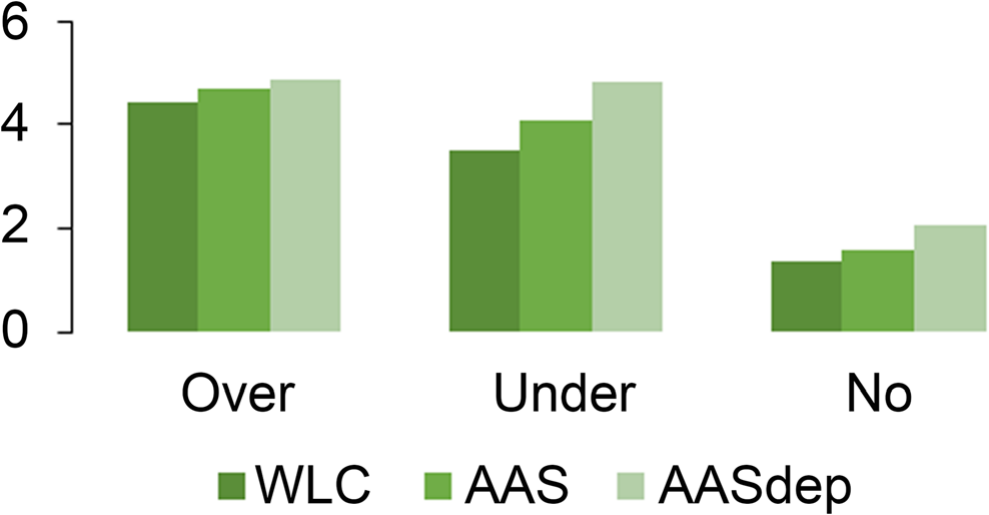
Number of overmentalizing, undermentalizing, and no mentalizing errors in individuals using anabolic androgenic steroids (AAS), individuals with anabolic androgenic steroid dependence (AASdep), and in weightlifting control participants (WLC)

In the repeated measures ANOVA for ToM errors, the main between-subject effect of group was significant [*F*_(2,173)_ = 5.50, *p* = 0.005, *ŋ* = 0.06], with post hoc Scheffe test showing again that the significant difference was between WLC and AASdep groups. Also again, both the main effect of sex [*F*_(2,173)_ = 0.09, *p* = 0.762, *ŋ* = 0.00] and the group x sex interaction [*F*_(2,173)_ = 0.76, *p* = 0.470, *ŋ* = 0.00] effect were non-significant. The main within-subject effect of ToM error was significant [*F*_(2,172)_ = 99.84, Wilk’s Lambda = 0.46, *p* < 0.001, *ŋ* = 0.54] (see Fig. 2).

No other interaction effects in any of the analyses were significant, see Table 3 for all results.

**Table 3:** Theory of mind performance in individuals using anabolic androgenic steroids (AAS), individuals with anabolic androgenic steroid dependence (AASdep), and in weightlifting control participants (WLC)

	WLC (*n* = 85)		AAS (*n* = 43)		AASdep (*n* = 51)		Statistics
	Females *n* = 16 Mean (SD)	Males *n* = 69 Mean (SD)	Females *n* = 9 Mean (SD)	Males *n* = 34 Mean (SD)	Females *n* = 7 Mean (SD)	Males *n* = 44 Mean (SD)	
Total ToM Range 0–45	36.9 (1.8)	35.4 (3.0)	34.1 (2.9)	34.7 (3.7)	32.9 (6.4)	33.3 (5.2)	*Group*: *F* = *5.6*, *p* = *0.004* *ŋ* = *0.06* Sex: *F* < 0.1, *p* = 0.839 *ŋ* = 0.00 G x S: *F* = 0.9, *p* = 0.430 *ŋ* = 0.01
Cognitive ToM Range 0–26	22.1 (1.5) 88%	21.4 (2.1) 82.3%	20.8 (2.3) 80%	21.0 (2.3) 80.8%	19.6 (4.0) 75.4%	20.1 (3.4) 77.3%	*Group*: *F* = *5.2*, *p* = *0.007* *ŋ* = *0.06* Sex: *F* = 0.2, *p* = 0.645 *ŋ* = 0.00 G x S: *F* = 0.6, *p* = 0.538 *ŋ* = 0.00
Affective ToM Range 0–18	14.4 (2.0) 80%	13.8 (1.8) 76.7%	13.7 (1.4) 76.1%	13.5 (2.1) 75%	13.1 (2.4) 72.8%	13.1 (2.2) 72.8%	
Overmentalizing errors Range 0–45	3.3 (1.5)	4.7 (2.6)	5.0 (3.4)	4.6 (2.9)	5.0 (5.5)	4.8 (2.5)	*Group*: *F* = *5.5*, *p* = *0.005* *ŋ* = *0.06* Sex: *F* = 0.1, *p* = 0.762 *ŋ* = 0.00 G x S: *F* = 0.8, *p* = 0.470 *ŋ* = 0.00
Undermentalizing errors Range 0–45	3.6 (1.4)	3.5 (1.9)	4.0 (1.6)	4.1 (2.4)	4.9 (1.9)	4.8 (2.7)	
No mentalizing errors Range 0–45	1.3 (1.3)	1.4 (1.2)	1.7 (1.0)	1.6 (1.3)	2.3 (1.1)	2.1 (1.6)	

Entries in italics means that a result is statistically significant

In the follow-up analysis controlling for the effect of drug use, the main effect of group on MASCtot remained significant [*F*_(6,157)_ = 4.29, *p* = 0.015, *ŋ* = 0.05]. It did not when IQ was controlled for [*F*_(6,178)_ = 2.78, *p* = 0.065, *ŋ* = 0.03]. The second follow-up analysis, of AAS users only, found no significant effect of being on or off cycle [*F*_(3,83)_ = 0.25, *p* = 0.616, *ŋ* = 0.01].

## Discussion

This study investigated the ToM abilities of males and females involved in heavy weightlifting who either did not use, who used, or who were dependent upon anabolic androgenic steroids. We found no sex differences, but a consistent pattern of group differences emerged. The AAS dependent group presented with worse performance than the other two groups, and significantly worse than the WLC group. The non-dependent AAS group had an intermediate performance between the other two groups but did not differ significantly from either. The same pattern was seen for the overall score, as for type of ToM (cognitive versus affective ToM) and for ToM errors (mentalizing styles). In other words, regardless of how ToM was assessed, individuals who were dependent on AAS performed worse.

Significant main effects were present for type of ToM (cognitive versus affective ToM) as well as for ToM error types. The significant main effect of type of ToM indicates that one type of ToM is easier to understand. Figure 1 shows the cognitive and affective ToM scores of the three groups, in percentage correct. MASCcog scores are better than MASCaff scores, for all three groups, suggesting that it is easier to understand others’ cognitions than their emotions, at least when using this test. Similarly, the significant main effect of ToM error types suggests that some errors are committed more often than others. The error scores of the three groups are depicted in Fig. 2; no mentalizing errors are fewer than the two other error types, regardless of group membership. These two significant main effects, yielded in two separate repeated measures ANOVAs, were not accompanied by significant interaction effects with group. This means that all three groups had a harder time understanding affective ToM than cognitive ToM, and that none of the groups committed a specific type of error more than the other two groups did.

The group difference for the MASC total score remained significant after controlling for levels of drug use, but not after controlling for IQ. This suggests that differences in our study variables may be explained by IQ, and that the group difference in ToM abilities is simply due to differences in IQ across the three groups. Studies that report significant associations between ToM and IQ can be taken as support for such an interpretation. In our sample, explorative correlational analyses showed that the association between MASCtot and IQ corresponded to a moderate-sized correlation coefficient (Spearman’s *rho* = 0.36). It was larger in the AAS (Spearman’s *rho* = 0.56) compared with the AASdep (Spearman’s *rho* = 0.30) and the WLC (Spearman’s *rho* = 0.18) samples. However, not only did the size of these correlation coefficients differ, ranging from small-medium to large, but also the association was not particularly strong in the group with the lowest IQ and worst ToM performance, the AASdep group. So, whereas it is a possibility that lower IQ may predispose individuals to AAS dependence as well as ToM impairment, these correlation coefficients imply that the poorer ToM abilities of the AASdep group cannot be reduced to their lower IQ. The effect of group on MASCtot after controlling for IQ (*p* = 0.065, *ŋ* = 0.03) aligns with this. It was not significant, but it approached trend-level significance. Moreover, a recent study that used the exact same tests in another clinical population suggests that impaired ToM is not redundant with IQ. In a sample of individuals with schizophrenia, WASI IQ was significantly associated with MASCtot (Spearman’s *rho* = 0.35) but did not provide a unique contribution when entered in a regression analysis (Sjølie et al. 2020). It therefore seems likely that our AASdep group indeed has selective ToM impairments, in addition to lower IQ. Importantly, “controlling” for group differences in potential covariates is not always appropriate (Miller and Chapman 2001). We believe that the identified group difference in ToM detected in this study has value in itself, providing meaningful information not conveyed by the diverging intellectual level of our three participant groups.

Interestingly, none of the analyses indicated that females differ from males. Admittedly, our study is underpowered with for instance only 7 females included in the AASdep group. However, exploratory follow-up analyses, conducted separately in each sex, confirmed significant group differences for MASC total in both males [*F*_(2,144)_ = 3.97, *p* = 0.021, *ŋ* = 0.05] and females [*F*_(2,29)_ = 3.75, *p* = 0.036, *ŋ* = 0.21]. Inspection of numerical values in Table 3 also supports the finding of no sex differences. One reason for the lack of statistical power is the relatively low number of females involved in weightlifting and use of AAS. Although AAS use is less common among females, there is historical evidence of its massive use, sometimes forced, in international sports. In the former German Democratic Republic (GDR), the administration of AAS to athletes was highly organized, overseen from the very top, and part of a political strive for success. Female athletes were certainly not spared, and sometimes received doses far exceeding what their male counterparts in the same sport were given (Franke and Berendonk 1997). Among side effects reported in GDR female athletes from this epoch were virilization (masculinized voice and facial features), hirsutism (excessive hair growth where hair does not normally grow such as outside pubic area towards navel/on thighs or in the face), and increased libido (Franke and Berendonk 1997). Less is known about cognitive side effects, but one hypothesis could be that females exposed to large amounts of AAS over time would experience a masculinizing effect on social cognition as well. Since it is often claimed that females have better social cognition than males, and there is empirical data in support of this position (Thompson and Voyer 2014), the lack of sex differences in our study could be taken to support such a hypothesis. However, as is the case for emotion recognition, identified sex differences in social cognition are often small and moderated by other factors (Thompson and Voyer 2014) or not present at all (Di Tella et al. 2020). Although there are studies that do report sex differences (Baron-Cohen et al. 2015), for ToM, the evidence of sex differences is even more limited (Turkstra et al. 2020), and an earlier study, using the exact same ToM measure, the MASC test, reported no sex differences for schizophrenia (Fretland et al. 2015). In spite of this, the public opinion is often that females are better mindreaders than males. One explanation for the lack of sex differences in the present study is that female weightlifters, regardless of whether they (ab)use AAS or not, do not have the alleged female advantage in the domain of social cognition. Whereas this is possible, it is perhaps just as likely that there are no sex differences in ToM, at least not when assessed with the MASC. In either case, the lack of significant sex and sex x group interaction effects may suggest that AAS do not exert any sex-specific effects on ToM, but clearly, more and larger studies are needed in order to fully answer this question.

There were no significant differences in ToM performance between AAS users who were currently on (on cycle: 43 participants) or off (off cycle: 40 participants) AAS. Whereas this may imply that levels of AAS are not of substantial importance to ToM, our study design does not allow for a sophisticated analysis of the association between ToM and specifics of AAS use. Longitudinal studies that administer ToM measures before, during, and after periods of AAS use would be better suited to answer such research questions.

When evaluating the level of social cognitive performance, it is necessary to move beyond comparisons with other weightlifters. It may be that the performance of our WLCs differs from those of healthy normal control participants or normative data. Unfortunately, such a group was not included in the current study. However, the performance of our WLC group (mean MASCtot: males = 35.4, WLC females = 36.9) was very similar to the performance of healthy controls (mean MASCtot = 35.1) in another Norwegian study (Vaskinn et al. 2018). This means that our AASdep sample has ToM impairments also when compared with non-weightlifting healthy controls.

The cross-sectional nature of this study renders us unable to provide evidence for any causal explanations for the reduced ToM seen in those dependent upon AAS. We would, however, like to suggest some possible explanations. The first is that the reduced ability to infer the mental state of others is related to long-term high-dose AAS use/dependence. A gradual alteration of the neuroendocrine and central nervous system may take place with prolonged AAS use, with negative effects on ToM abilities. The fact that AAS users without dependence had an intermediate performance between non-using weightlifters and dependent AAS users is in line with this explanation, as are previous reports stating that pronounced adverse effects of AAS are foremost seen after long-term exposure and in dependence (Copeland et al. 2000; Kanayama et al. 2009b). Another explanation is that a vulnerability for developing substance use disorder co-exists with premorbidly lower social cognition in the AASdep group.

Our study has several limitations. We have mentioned the cross-sectional design and the lack of a non-weightlifting healthy control group. In addition, the number of female participants is much lower than the number for male participants. Although there are obvious reasons for this, ideally, at least scientifically speaking, we would have wished for a larger female sample. Furthermore, exact measurements of testosterone levels were not available. How current testosterone levels relate to ToM is therefore unknown. Moreover, we acknowledge that factors that we have not assessed, for example, childhood experiences and other clinical variables, may be of relevance to the association between AAS use and ToM.

In summary, we found that male and female weightlifters who were dependent on AAS had reduced ToM abilities compared with non-using weightlifters. The reduced ToM may be among the factors underlying the higher rates of antisocial behavior reported in this population.
